# Evolution and Function of Thioester-Containing Proteins and the Complement System in the Innate Immune Response

**DOI:** 10.3389/fimmu.2017.00759

**Published:** 2017-06-29

**Authors:** Upasana Shokal, Ioannis Eleftherianos

**Affiliations:** ^1^Department of Biological Sciences, The George Washington University, Washington, DC, United States

**Keywords:** insects, mammals, innate immunity, thioester-containing proteins, complement system, *Drosophila*, mosquito

## Abstract

The innate immune response is evolutionary conserved among organisms. The complement system forms an important and efficient immune defense mechanism. It consists of plasma proteins that participate in microbial detection, which ultimately results in the production of various molecules with antimicrobial activity. Thioester-containing proteins (TEPs) are a superfamily of secreted effector proteins. In vertebrates, certain TEPs act in the innate immune response by promoting recruitment of immune cells, phagocytosis, and direct lysis of microbial invaders. Insects are excellent models for dissecting the molecular basis of innate immune recognition and response to a wide range of microbial infections. Impressive progress in recent years has generated crucial information on the role of TEPs in the antibacterial and antiparasite response of the tractable model insect *Drosophila melanogaster* and the mosquito malaria vector *Anopheles gambiae*. This knowledge is critical for better understanding the evolution of TEPs and their involvement in the regulation of the host innate immune system.

## Introduction

Innate immunity is a fundamental process for early recognition and subsequent induction of proinflammatory responses against invading pathogens (1). Insects are outstanding models for studying innate immune functions and host–pathogen interactions (2–4). Insects activate a variety of innate immune responses depending upon the type of pathogen they encounter. The cell signaling machinery involved in the insect innate immune response is structurally and functionally similar to innate immune pathways in mammals (5, 6). Previous and recent research involving infections with bacterial and fungal pathogens has led to the identification and characterization of two distinct immune pathways, the toll pathway [similar to mammalian IL-1/TLR pathway (7)] and the Immune deficiency pathway [Imd, similar to mammalian TNF-αR signaling pathway (8)], which regulate NF-κB transcription factors that control the expression of several antimicrobial peptide (AMP) coding mainly in the fat body tissue (9). In addition, the Janus kinase/signal transducer and activator of transcription (JAK/STAT) and c-Jun N-terminal kinase (JNK) signaling pathways also act in either competing or cooperative modes to modulate the activity of immune effector genes (10, 11).

Insects utilize germ line-encoded receptors known as pathogen recognition receptors (PRRs) to identify distinct pathogen-associated molecular patterns (PAMPs) that are either present on the surface of microbial pathogens or are released in the host during the infection (12). Insect PRRs are classified into three classes—secreted, endocytic, and signaling (13). A special class of signaling PRRs in the fruit fly *Drosophila melanogaster* is the peptidoglycan recognition proteins (PGRPs) (14). PGRP-SA and PGRP-SD bind to Gram-positive bacteria and activate a protease cascade that induces the toll signaling pathway (15, 16). PGRP-LE and PGRP-LC recognize DAP-type peptidoglycan structures present on the Gram-negative bacteria (17). To identify fungal pathogens, PRRs such as Gram-negative binding protein-3 target the β-(1,3)-glucan structure present on the fungal cell wall (18). Binding of these proteins to their molecular targets results in downstream activation of the NF-κB signaling pathways Imd and toll (19). In addition to the signaling PRRs, insect genomes also contain secreted recognition molecules such as the thioester-containing proteins (TEPs), named after their active site that functions by forming covalent bonds with specific molecular targets (20). This mini review describes the complement proteins in mammals and the participation of TEPs in the immune response of mosquitoes and flies.

## Thioester-Containing Proteins

Members of the TEPs family have been recognized in primitive Protostomes and in Deuterostomes, ranging from *C. elegans* to mammals. TEPs contain a thioester (TE) motif, GCGEQ, which includes a highly unstable covalent bond between the side groups of cysteine and nearby glutamic acid (21). These proteins remain inactive in the native state due to a shielded environment within the protein, but when they encounter elevated temperature, aqueous conditions, or undergo proteolytic activation; the TE bond becomes active for a very short time (22–24). The active TE motif has the ability to bind to nearby accessible hydroxyl and amine groups that are present on all biological surfaces including pathogens (25). TEPs are classified into two subfamilies—complement factors and alpha-2 macroglobulins (α-2Ms). Once activated, the complement factors produce a small anaphylatoxin fragment lacking the TE motif and a larger fragment that binds to the target as a result of hydrolysis of the TE bond (20). The small anaphylatoxins act as immunoinflammatory stimulators and chemoattractants that recruit macrophages to the infection site. The larger, covalently bound fragment marks the pathogen as foreign and targets it for lysis or phagocytosis. In contrast, the α-2Ms inhibit the protease activity of pathogens *via* a conformational change that traps the attacking protease after linkage with the TE motif within the protein. This conformational change also exposes the receptor-binding domain of the α-2Ms that promotes receptor-mediated endocytosis for clearance of the pathogen through physical interaction with cell surface receptors (26). Hence, both complement factors and α-2Ms serve important functions in recognition as well as clearance of the pathogens from the host. Certain TEPs such as *Drosophila* TEP6, C5 in higher vertebrates, and ovostatin in mammals, contain a mutated TE motif (27). It has been further suggested that the presence of certain TEPs in the host could be an outcome of different environments, selective pressures, and perhaps gene duplications events (28, 29). Functional characterization of TEPs in model organisms would shed light on their importance and specificity in the host.

## Complement Proteins in Mammals

The complement system is an important effector that functions at the intersection of innate and adaptive immune responses in mammals. The system includes 50 germ line-encoded, circulating, and membrane-bound proteins. The activation of the complement system triggers a protease cascade that ends in opsonization and/or lysis of the pathogen. In addition to being pro-inflammatory, the complement proteins are also involved in homeostatic processes such as removal of dying cells with exposed danger-associated molecular patterns (DAMPs) that consequently generate a sterile inflammatory reaction (30, 31). In certain cases, activation of the complement cascade results in host tissue damage leading to autoimmune and chronic inflammatory diseases (32). Hence, host molecules closely control the activation and regulation of complement system.

**Figure 1:**
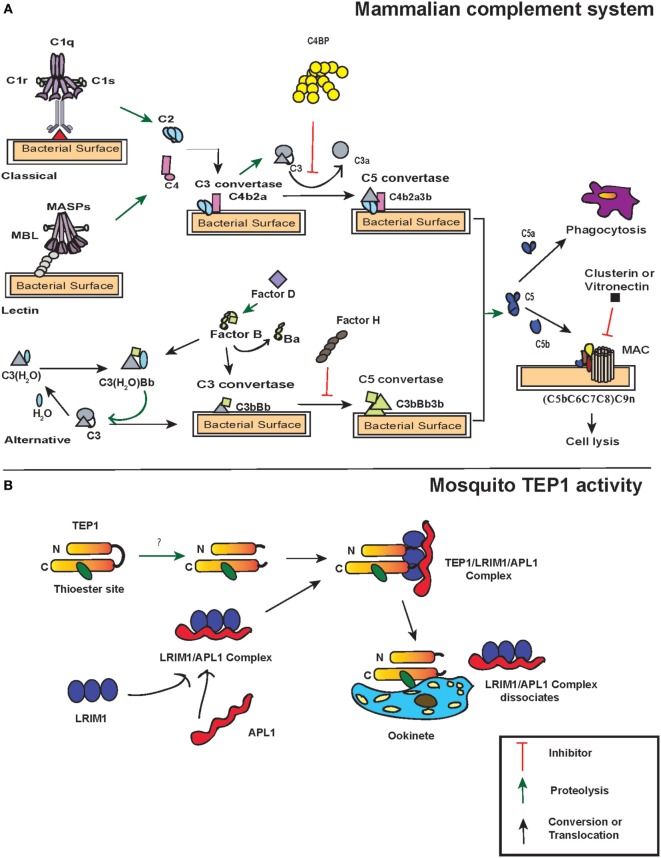
Action of thioester-containing proteins (TEPs) in mammals and mosquitoes. **(A)** Pattern recognition receptors identify the presence of pathogens. In the classical pathway, C1 complex (specifically C1q) recognizes pathogen-associated molecular patterns or danger-associated molecular patterns either through binding to them directly or through binding of antibodies to the foreign antigen. This activates C1r that subsequently leads to C1s activation. Similarly, in the lectin pathway, binding of mannan-binding lectin (MBL) activates MBL-associated serine protease (MASP-1) and MASP-2. Activation of C1 and/or MBL–MASP complex leads to the cleavage of C4 and C2 molecules into C4a, C4b, C2a, and C2b, subsequently forming C3 convertase (C4b2a) that binds to the microbial surface. The newly formed C3 convertases cleave C3 into C3b that also binds to the microbial surface. Bound C3b recruits Factor D that activates Factor B, which results in the formation of C3bBb (C3 convertase of alternative pathway). C3bBb cleaves more C3 and initiates an amplification loop. Additionally, a fluid-phase convertase could also be formed when water associates with C3, forming C3(H_2_O). The latter reacts with activated Factor B and thus maintains a low level of complement activation known as tick-over mechanism. The C3 convertases generated from each pathway bind to C3b forming C5 convertase, which cleaves C5 into C5a and C5b. The latter initiates the formation of membrane attacking membrane by recruiting C6, C7, C8, and C9 complement proteins. Certain molecules such as C4-binding protein, Factor H, vitronectin, and clusterin act as regulators of complement proteins. **(B)** TEP1 is constitutively activated in the hemolymph by one or more unknown proteases. The proteolytic cleavage produces two fragments TEP1-N and TEP1-C that remain associated with each other. Two leucine-rich repeats (LRRs) proteins, LRIM1 and APL1, maintain the mature form of TEP1. Upon recognition of the parasite, TEP1 dissociates from the LRR proteins by yet an unknown mechanism and binds to the parasite, which ultimately leads to its destruction. Arrows represent inhibition (red), proteolytic cleavage (green), and conversion or translocation of a molecule (black).

The activation of complement system in mammals is regulated through three distinct pathways: the classical pathway, the lectin pathway, and the alternative pathway. Although these pathways have different ligands and receptors, they all converge to produce the same sets of effector molecules (33) (Figure 1A). The initiation of the classical pathway occurs upon binding of the collectin type PRR C1 complex (C1q multimers with inactive serine proteases C1r and C1s) to an antigen–antibody complex, to PAMPS, or to DAMPs (34–36). When C1q binds to PAMPs, a conformational change occurs in C1r and C1s complex, which results in autocatalytic activation of C1r. The activated C1r serine proteases then activate the C1s, which in turn cleave C4 and C2 molecules into the small anaphylatoxin C4a or C2b and the larger C4b or C2a, respectively. This exposes the activated TE within C4b, which binds covalently to the pathogen surface and recruits C2a to form the C4b2a complex. This newly formed complex on the pathogen surface is a C3-convertase that will perpetuate the cascade.

Similar to the classical pathway, the lectin pathway PRRs, either mannan-binding lectin (MBL) or ficolins L/M/H (ficolins-L or ficolins-M or ficolins-H) recognize specific sugars or acetylated moieties on the surfaces of Gram-positive bacteria, Gram-negative bacteria, fungi, protozoans, and viruses (37–39). The lectin pathway PRRs form complex with two MBL-associated serine proteases (MASP)-1 and MASP-2 that are structural homologs of C1r and C1s (40). Thus, MASP-1 and MASP-2 react and cleave C4 and C2 molecules to form the same C3-convertase, as described for the classical pathway. In contrast to the classical and lectin pathways, the alternative pathway does not require pathogen recognition proteins for its activation. Instead, it is initiated through spontaneous generation (also called tick-over mechanism) of short-lived C3(H_2_O) by hydrolyzing the TE bond in the C3 molecule. This short-lived molecule binds to factor B in solution, which causes a conformational change in the structure of factor B. This leads to the cleavage of factor B into Ba and Bb fragments by factor D forming the C3(H_2_O)Bb complex, which is the alternative pathway version of a C3-convertase, also called fluid-phase C3-convertase.

The C3-convertases produced by each of the three pathways generate C5-convertases upon binding C3b to C4b2b in the classical and lectin pathways yielding C4b2b3b. The alternative pathway C3-convertases can cleave many molecules of C3 into C3a and C3b. While most of the C3b is inactivated by hydrolysis, a fraction is able to link covalently to the PAMPs through the TE bond and form C3b2Bb (C5 convertases). The C5-convertases act on C5 and cleave it to C5a and C5b. C5a is released as an anaphylatoxin, and C5b recruits complement factors C6, C7, C8, and C9 that form the membrane-attack complex (MAC) in the cell membrane of the pathogen. While the larger fragment C5b plays a central role in MAC formation, the shorter C5a fragment acts on the endothelial or mast cells and increases the permeability of the blood vessels as well as extravasation of immunoglobulins to the site of inflammation. The activity of C5a causes a septic-shock state called anaphylactic shock and eventually triggers the inflammatory response. Together, these molecules assist in recognition, opsonization, and phagocytosis or lysis of pathogens, and are involved in the activation of adaptive immunity in vertebrates (41, 42) (Figure 1A).

The complement factors with TE motifs can also bind self-molecules containing accessible hydroxyl or amine groups on their surface. Therefore, to avoid false activation of the complement cascade in the absence of foreign entities, several complement regulatory proteins are present in mammals. One of the most potent and well-studied regulatory proteins is complement factor H that initiates the decay of the C3-convertase complex by dissociating Bb from C3b (43). Factor H competes with the Bb fragment and binds to C3(H_2_O), which results in the dissociation of factor B from the latter. Moreover, it can bind host-specific glycans to prevent complement activation on host surfaces (44). Another regulator is the C4-binding protein (C4BP) that regulates the classic and lectin pathways with similar activities as factor H by targeting C4b and C2a (45). C4BP acts as a decay-accelerating factor and dissociates C2a from the C3-convertases. While these regulators control the formation of C3-convertase, other complement regulators such as clusterin and vitronectin inhibit MAC assembly or C9 insertion into membranes after the formation of C3 convertase complex and activation of the terminal pathway (46, 47).

Although the complement system is extremely efficient in fighting and clearing pathogenic infections, certain bacterial and viral pathogens can evade this immune response (48, 49). They achieve this by escaping the complement action through binding to the complement inhibitors, which target active complement factors that interfere with MAC complex formation and mimic host surfaces (50–52).

## TEPs in Insects

**Figure 2:**
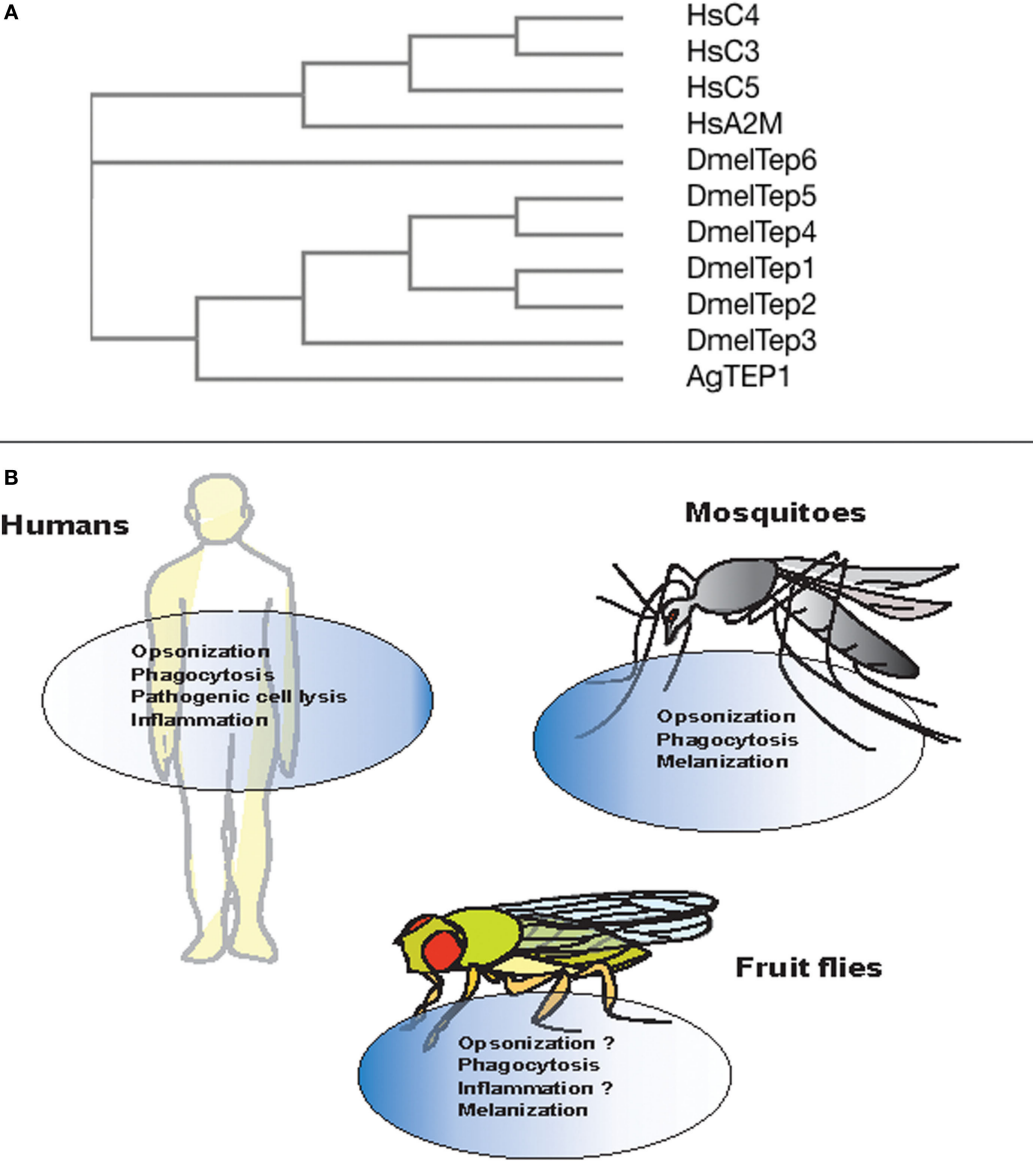
Evolution and conserved function of thioester-containing proteins (TEPs) in mammals, mosquitoes, and fruit flies. **(A)** Phylogenetic tree showing the evolution and similarity between different complement factors, alpha-2 macroglobulin in *Homo sapiens* (Hs), and TEPs in *Anopheles gambiae* (Ae) and *Drosophila melanogaster* (Dm), generated through Clustal-Omega. **(B)** Complement proteins are involved in immune functions such as opsonization, phagocytosis, cell lysis of pathogens, and act as pro-inflammatory molecules. Similarly in mosquitoes, TEP molecules participate in the opsonization, phagocytosis, and melanization of bacterial pathogens and *Plasmodium* parasites. However, with the exception of phagocytosis and melanization, immune anti-pathogen activities of TEPs are yet to be identified in the fly.

Phylogenetic analysis of TEP-coding genes in dipteran insects, other invertebrates, and vertebrate animals has classified them into three subfamilies including complement factors, α-2Ms, and insect TEPs (20) (Figure 2A). The complement factor subgroup containing C3, C4, and C5 proteins is the most fast-evolved TEP subfamily. On the other hand, the α-2Ms are present in a larger group of animals other than the two subfamilies, which suggests their slow evolution due to several functional constraints on the structure of these inhibitors (53). Insect TEPs are highly diverged as well as unstable, and they are more related to the α-2M family than to the TE complement factor group (20) (Figure 2A). The presence of multiple TEP homologs in mosquitoes relative to those in *Drosophila* indicates that different adaptations between these insects have led to gene duplication and the generation of more homologs (54). It is currently unknown whether regulators of TEPs, such as homologs of human C4BP or Factor H, in insects exist. Interestingly, mosquitoes can capture Factor H from ingested human blood to escape the deleterious effects of the complement activation system (55). Although there is high structural and functional homology between TEPs and complement proteins, it is unclear whether insect TEPs possess a mechanism of action similar to C3 tick over. Here, we summarize TEPs in mosquitoes and fruit flies.

### Mosquito TEPs

Genome sequencing of two mosquito species, *Anopheles gambiae* and *Aedes aegypti*, has contributed toward understanding several molecular mechanisms involved in host immunity. Various components of the complement pathway, specifically, complement-like proteins have been identified in the two genomes. The *A. gambiae* genome contains 19 TEP gene homologs (*AgTep* 1–19), of which four pairs show haplotypic features (*AgTep1–AgTep16, AgTep5–AgTep17, AgTep6–AgTep18, and AgTep7–AgTep19*) and hence, represent polymorphic variations rather than distinct genes (54, 56). There are eight *Tep* genes in *A. aegypti* (AeTEP 1–8) encoding TEP proteins that share 21–39% amino acid similarity to *AgTEP1* (57, 58). In addition, the mosquito TEPs share structural and functional similarities with mammalian α-2Ms (29, 59).

A key immune gene identified through functional studies in *A. gambiae* was *AgTep1*. AgTEP1 is a constitutively secreted hemolymph protein with a size of 165 kDa (TEP1-full) and its cleavage results in the formation of an 80 kDa active fragment (TEP1-cut) (60). While the N-terminal region of AgTEP1 has a hydrophobic signal peptide-like segment as well as a canonical TE motif plus a catalytic histidine residue that is positioned 100 amino acids downstream, the C-terminal region has a cysteine signature. The TEP1-cut circulates in the hemolymph in association with two leucine-rich repeats (LRRs) proteins, LRIM1 and APL1C (61, 62). These two LRR proteins act as TEPs regulators and promote pathogen recognition as well as their destruction (Figure 1B).

Several studies have shown the functional importance of AgTEP1 in various processes such as recognition, opsonization, and phagocytosis of certain bacteria. *In vitro* and *in vivo* studies have shown that bacteria are phagocytosed when the C-terminal part of AgTEP1, also called AgTEP1-cut, binds to bacteria (63). Moreover, knockdown of *AgTep1* or culturing of hemocytes with methylamine-treated conditioned medium (prevents autocatalytic fragmentation of the full-length protein into smaller 80 kDa cut fragment) reduced the efficiency of phagocytosis of Gram-negative bacteria by 50–75% (63). Another study also showed decrease in phagocytosis of *Escherichia coli* and *Staphylococcus aureus* after the depletion of *AgTep1* expression (62). Thus, AgTEP1 acts as an opsonin and marks targeted bacteria for phagocytosis (Figure 2B).

The complement C3-like protein, AgTEP1, is an important molecule in inducing an immune response against *Plasmodium berghei*. The protein binds to the surface of the parasite and triggers its encapsulation by hemocytes, which leads to parasite death. Two divergent alleles of *AgTep1-AgTep1r* and *AgTep1s* are reported (64). While *AgTep1s* is present in most mosquito populations making them susceptible to *P. berghei* infection, the allele *AgTep1r* confers high resistance to the same parasite (65). Moreover, silencing of *AgTep1* also inhibits parasitic lysis and actin polymerization (66). The regulatory molecules LRIM1 and APL1 are also required for binding of AgTEP1 to the parasites (61). The LRIM1 and APL1 complex not only interacts with AgTEP1 but also interferes with three other TEP proteins, including AgTEP3 (64). Silencing the two genes encoding the LRR proteins results in the conversion of refractory strains into susceptible strains (61, 63).

Although several functional studies have been performed on AgTEPs, only a few studies have characterized the specific function of *A. aegypti* TEPs. The genes *AeTep1–AeTep5* are all constitutively expressed throughout the body of adult mosquitoes. A study on *A. aegypti* has shown a twofold to threefold increase in West Nile virus load after silencing the *AeTep1* and *AeTep2* genes whereas overexpression of *AeTep1* and *AeTep3* resulted in a decrease in viral load (58). Thus, AeTEPs has an important function in the mosquito host defense by limiting viral infection.

The mosquito TEPs have been found to possess conserved function similar to complement factors by binding to bacteria or *Plasmodium* parasites, which results in the phagocytosis of bacteria as well as melanization and lysis of the parasites, respectively (59) (Figure 2B). Future studies will focus on investigating the binding specificity of mosquito TEPs and how the binding process leads to parasite lysis at the molecular level.

### Fruit Fly TEPs

In insects, TEPs were first discovered in *Drosophila melanogaster* (67). While there is a plethora of information on mosquito TEPs, there are only few studies on the immune function of TEPs in *Drosophila*. The *D. melanogaster* genome contains six TEP homologs (68). TEP1-4 contain a conserved TE motif. *Tep5* may represent a pseudogene as it is found in the genomic sequences but is not expressed (13). *Drosophila* TEPs have a highly conserved region of 30 amino acids that compose the N-terminal of the TE motif, a cysteine signature tail, which is similar to *Anopheles* TEPs. They also have a 60 amino acid hypervariable region, which is structurally similar to mammalian bait region of α-2Ms as well as to the anaphylatoxin domain in vertebrate C3b (67). TEP6 is the only TEP that lacks a functional TE motif, exhibiting a serine instead of cysteine residue. Of the six *Tep* genes, only *Tep2* shows alternative splicing in exon 5 producing five different isoforms. The alternative splicing occurs in the exon region that codes for the hypervariable domain of the TEP2 protein. The alternative splicing may aid in increasing the inhibitor proteases repertoire and augmented diversity of recognition receptors. Flies may have evolved a strategy to encounter distinct pathogens that is analogous to VDJ diversity generated by adaptive immunity in higher vertebrates (69).

*Drosophila melanogaster Teps* are upregulated in different tissues and participate in immune response and developmental processes. *Teps* are expressed in larval hemocytes, fat body, and in the gut barrier epithelia, whereas, in the case of adults, *Teps* are expressed in the fat body of the head, spermatozoa, and midgut epithelia in the absence of infection (70). Upon bacterial challenge, *Tep1, Tep2*, and *Tep4* are upregulated in *D. melanogaster* larvae, whereas only *Tep1, Tep2, Tep4*, and *Tep6* are upregulated in adults in response to certain bacterial, fungal, or parasitoid infection (67, 70–72). Additionally, *Tep2* and *Tep3* are upregulated against parasitic infections with the nematode *Heterorhabditis bacteriophora* that contains the mutualistic bacteria *Photorhabdus luminescens* (73). Loss-of-function *tep2* and *tep4* mutants are susceptible to *Pseudomonas ginigivalis* infection whereas loss-of-function *tep3* mutants are susceptible to *H. bacteriophora* infection (74, 75). Another study reported that *tep1–4* mutant flies were slightly resistant to bacterial infection in comparison to wild-type flies (69). Although these studies reported the involvement of fly TEPs in the antibacterial and antiparasitic immune response, the mechanism of TEPs action was not clarified. More recently, it was shown that TEP2, TEP4, and TEP6 has an important regulatory role in the innate immune response of *D. melanogaster* adult flies against the pathogenic bacteria *Photorhabdus* (72, 76). *Tep2, Tep4*, and *Tep6* are transcriptionally upregulated in response to *P. luminescens* and *P. asymbiotica* infection. Moreover, transcriptional activation of these genes influences the activation of toll, Imd, JAK/STAT, and JNK signaling and results in differential expression of AMP and stress coding genes. *Tep2* and *Tep4* upregulation also decreases phenoloxidase activity and the melanization response during the early stages of *Photorhabdus* infection. As a result, these effects promote the survival of flies upon infection with pathogenic *Photorhabdus*. This is the first evidence of the involvement of a TEP in the fly antibacterial immune system.

*In vitro, D. melanogaster* TEP2, TEP4, and TEP6 (MCR or macroglobulin-complement related) promote phagocytosis of certain Gram-negative bacteria and fungal pathogens (77). The rate of phagocytosis in *D. melanogaster* S2 cells incubated with *Candida albicans* decreases upon *Mcr* silencing because the MCR protein binds specifically to the fungal surface. Moreover, the rate of *E. coli* and *S. aureus* phagocytosis is reduced after silencing *Tep2* and *Tep4* genes. In addition, inactivation of *Tep2* and *Tep6* significantly impairs the expression of *Eater* gene in adult flies suggesting that TEP2 and TEP6 participate in the phagocytic response against *Photorhabdus* bacteria (72) (Figure 2B). This suggests that different TEP molecules are involved in the immune response and probably recognition of different pathogens. It has been suggested previously that JAK/STAT and toll pathways regulate the expression of TEP1, but the mechanisms are poorly understood (67, 78).

## Concluding Remarks and Future Prospects

Recent efforts have mostly focused on understanding the molecular and genetic mechanisms that regulate the participation of TEPs in interfering with the transmission of eukaryotic parasites and activating innate immune responses against pathogenic infections in insects (76, 79–82). Future studies could potentially examine the tissue-specific patterns of induction of insect *Tep* genes upon infection with different pathogenic and non-pathogenic microorganisms. Tissue-specific profiling of *Tep* gene expression would possible denote their specificity toward certain microbial infections. For example, the upregulation of *Tep* genes in the fat body, gut, or hemocytes upon microbial challenge would indicate their involvement in the insect humoral and/or cellular immune response to microbial invaders. Indeed, complement proteins are involved in the activation the humoral immune response in invertebrates and vertebrates. In mammals, complement factors are involved in the regulation of humoral immune responses (83). In insects, complement-related factors participate in the upregulation of AMPs against flavivirus infection in the mosquito, *A. aegypti* (84). Recently, it has been proposed that macrocapsules loaded with α-2Ms enhance certain human leukocyte functions, such as the recruitment of leukocytes to the site of inflammation and phagocytosis (85). Although TEP1 is involved in opsonization and phagocytosis of certain bacteria in mosquitoes (20, 26), other TEP molecules, might also participate directly or indirectly in insect cellular immune processes.

In addition in mammals, there is an intricate cross talk between the complement system and the coagulation cascade (86). Within certain hours of pathogenic infection, both of these systems are activated through the activity of serine proteases (87). Likewise, the coagulation system and the phenoloxidase cascade are linked in insects (88). It has been shown that *A. gambiae* TEP1 is essential in the process of melanization of *Plasmodium* parasites (6), and phenoloxidase activity as well as the melanization response are affected in *Drosophila* flies inactivated for *tep2* and *tep4* genes when responding to the pathogen *Photorhabdus* (72, 76) (Figure 2B). Future research could concentrate on the identification of the molecular components that facilitate the interaction between complement and coagulation systems in vertebrates and invertebrates.

Complement proteins are involved in the inflammation process and programmed cell death in vertebrates (89, 90). The presence of complement serves a protective function in vertebrates, but complement activation can also be deleterious for the host (91). Deletion in C5a confers resistance and reduced bacteremia shock in mice in response to Gram-negative bacterial infection (92). Identification of TEPs in insects with function analogous to C5a in mammals or relevance to pathophysiological defects in the host offers an exciting and challenging area of future research. In conclusion, future studies on elucidating the molecular mechanisms of interaction of TEPs with specific host physiological processes will undoubtedly shed light on their exact anti-pathogen immune function as well as their evolution in the animal kingdom.

## Author Contributions

US wrote the paper and IE revised it.

## Conflict of Interest Statement

The authors declare that the research was conducted in the absence of any commercial or financial relationships that could be construed as a potential conflict of interest.
